# Supplementary material for: Content-rich biological network constructed by mining PubMed abstracts
Source: BMC Bioinformatics. 2004 Oct 8;5:147. doi: 10.1186/1471-2105-5-147 (PMC528731; doi:10.1186/1471-2105-5-147)
Supplement: Additional File 2 — The original results of the above study (non-essential files are deleted to keep the file size under the limit set by BMC bioinformatics). [file 1471-2105-5-147-S2.bz2 › chilibotAdditionalFile2/dip05/227ID7954478E744/html/CTNNB1_CTNNA1.html]

 


 **CTNNB1** and **CTNNA1** 
  
Found 3187 abstracts in PubMed, retrieved 05.  
 

 What does Google say? 
 PDF only 
| .edu only 

---

**Interactive relationship** (e.g. stimulation, inhibition, etc)

**Non-interactive relationship** (e.g. studied together, co-existance, homology, etc.)

- TGF beta1 mediated MAP kinase activation also controls Wnt 7a gene expression and Wnt mediated signaling through the intracellular beta catenin  [ **CTNNA1** ]   [ **CTNNB1** ]  TCF pathway, which likely regulates N cadherin expression and subsequent N cadherin mediated cell adhesion complexes during the early steps of MPC chondrogenesis.  Ref: 12893825 J Biol ChemJ Biol Chem,
- Src activity was increased in QLalpha12 MDCK cells as assessed by Src autophosphorylation, and beta catenin  [ **CTNNA1** ]   [ **CTNNB1** ]  tyrosine phosphorylation was also increased, although there was no detectable increase in Rho activity.  Ref: 12890651 Am J Physiol Cell PhysiolAm J Physiol Cell Physiol,
- Western blotting was used to determine polypeptide size and expression of E cadherin and beta catenin  [ **CTNNA1** ]   [ **CTNNB1** ]  in Wilms s tumours compared with normal kidney.  Ref: 12890743 Mol Pathol, 2003
- Low to high gamma catenin  [ **CTNNA1** ]   [ **CTNNB1** ]  and ezrin expression was seen in cytoplasm of blastemal and epithelial cells of primary and metastatic tumours.  Ref: 12890743 Mol Pathol, 2003
- CONCLUSIONS Compared with primary tumours, metastatic tumours showed lower expression of E cadherin and gamma catenin  [ **CTNNA1** ]   [ **CTNNB1** ] , with nuclear staining for beta catenin  [ **CTNNA1** ]   [ **CTNNB1** ] .  Ref: 12890743 Mol Pathol, 2003
- We have studied the effects of the organophosphorous pesticide malathion on cell viability, actin cytoskeleton, cell adhesion complex E cadherin beta catenin  [ **CTNNA1** ]   [ **CTNNB1** ] , and Rho and Rac1 GTPases from the human mammary carcinoma cell line MCF 7.  Ref: 12888906 Int J Oncol, 2003
- AIMS To evaluate E cadherin, beta catenin  [ **CTNNA1** ]   [ **CTNNB1** ] , gamma catenin  [ **CTNNA1** ]   [ **CTNNB1** ] , and ezrin expression and its relation to histopathological features of primary and metastatic Wilms s tumours.  Ref: 12890743 Mol Pathol, 2003
- BACKGROUND The E cadherin catenin  [ **CTNNA1** ]   [ **CTNNB1** ]  adhesion complex is crucial for intercellular adhesiveness and maintenance of tissue architecture.  Ref: 12890743 Mol Pathol, 2003
- Primary and metastatic tumours showed moderate to high beta catenin  [ **CTNNA1** ]   [ **CTNNB1** ]  expression in blastemal and epithelial cells, with predominantly membranous and cytoplasmic staining.  Ref: 12890743 Mol Pathol, 2003
- beta Catenin  [ **CTNNA1** ]   [ **CTNNB1** ]  mutations in liver tumors induced by 2 amino dimethylimidazo f quinoline in CDF 1 mice.  Ref: 12893427 Cancer Lett, 2003
- These changes were related to a decrease in the expression of the adhesion molecules, E cadherin and beta catenin  [ **CTNNA1** ]   [ **CTNNB1** ] , and to the distribution and reactivity of actin microfilaments to TRITC phalloidin.  Ref: 12888906 Int J Oncol, 2003
- In this study, we screened liver tumors induced by 2 amino dimethylimidazo f quinoline MeIQ in CDF 1 mice for beta catenin  [ **CTNNA1** ]   [ **CTNNB1** ]  and APC mutations and other genetic alterations shown to occur in human hepatocellular carcinomas HCC, including mutations in the p53 and H ras genes, c myc amplification and E cadherin promoter methylation.  Ref: 12893427 Cancer Lett, 2003
- METHODS Immunohistochemistry was used to determine the expression and cellular distribution of E cadherin, beta catenin  [ **CTNNA1** ]   [ **CTNNB1** ] , gamma catenin  [ **CTNNA1** ]   [ **CTNNB1** ] , and ezrin in primary and metastatic Wilms s tumours.  Ref: 12890743 Mol Pathol, 2003
- These data indicate that except for the occasional disruption of the Wnt pathway through beta catenin  [ **CTNNA1** ]   [ **CTNNB1** ]  mutations, the genetic pathways involved in the development of HCC differ significantly between human liver cancer and tumors induced in mice by MeIQ, but do not rule out the possibility that heterocyclic amines constitute a carcinogenic risk factor in humans.  Ref: 12893427 Cancer Lett, 2003
- Higher amounts of 92 kDa beta catenin  [ **CTNNA1** ]   [ **CTNNB1** ]  were detected in tumours than in normal kidney.  Ref: 12890743 Mol Pathol, 2003
- Differential expression of E cadherin and beta catenin  [ **CTNNA1** ]   [ **CTNNB1** ]  in primary and metastatic Wilms s tumours.  Ref: 12890743 Mol Pathol, 2003
- SSCP followed by direct DNA sequencing revealed mutations in exon 2 of the beta catenin  [ **CTNNA1** ]   [ **CTNNB1** ]  gene in 2 of 16 liver tumors 12.5%.  Ref: 12893427 Cancer Lett, 2003
